# Ready-to-Use or Ready-to-Adapt: Can the Self-Healing Potential of *Bacillus licheniformis* Be Modified?

**DOI:** 10.3390/bioengineering13050495

**Published:** 2026-04-24

**Authors:** Luka Mejić, Olja Šovljanski, Milada Pezo, Lato Pezo, Tiana Milović, Ana Tomić

**Affiliations:** 1 Faculty of Technical Sciences, University of Novi Sad, Trg Dositeja Obradovića 6, 21000 Novi Sad, Serbiatiana.milovic@uns.ac.rs (T.M.); 2 Faculty of Technology Novi Sad, University of Novi Sad, Bulevar cara Lazara 1, 21000 Novi Sad, Serbia; 3 “Vinča” Institute of Nuclear Sciences, University of Belgrade, Mike Petrovića Alasa 12, 11000 Belgrade, Serbia; 4 Institute of General and Physical Chemistry, Studentski Trg 12-16, 11000 Belgrade, Serbia

**Keywords:** *Bacillus licheniformis*, microbial self-healing concrete, microbiologically induced calcium carbonate precipitation, UV mutagenesis, biomineralization, cementitious materials, data-driven approach, artificial neural networks, predictive modeling tools

## Abstract

In recent years, bacteria-based self-healing has emerged as a promising bioengineering strategy to address the self-repair of cracks in cement-based materials, which represent one of the persistent durability challenges. This approach relies on microbiologically induced calcium carbonate (CaCO_3_) precipitation (MICP), in which metabolically active bacteria promote CaCO_3_ formation of crystals that can heal cracks and restore material integrity. This study compares the self-healing potential of a natural (N-) alkaline soil *Bacillus licheniformis* strain with a UV-strain (phenotypic mutant) generated through controlled UV exposure followed by adaptive evolution. Both strains were evaluated under conditions relevant to cementitious environments. The UV-strain exhibited enhanced ureolytic performance, reaching urease activity of 0.32 U/mg compared to 0.24 U/mg in the N-strain. This translated into improved biomineralization, with CaCO_3_ precipitation reaching 2.37 mg versus 2.23 mg/100 mL in the N-strain. Additionally, the UV-strain showed increased cell hydrophobicity and aggregation, indicating improved nucleation potential and surface-mediated mineral deposition. Multivariate analysis confirmed strong correlations between ureolytic metabolism, alkalization, and mineral formation, while artificial neural network (ANN) modeling (MLP 6-10-14) successfully predicted biomineralization-related parameters with high accuracy (R^2^ > 0.90 for urease activity, NH_4_^+^, ΔpH, and CaCO_3_). The results demonstrate that UV-induced phenotypic adaptation can enhance biomineralization efficiency with minor trade-offs in physiological robustness. For the first time, that controlled UV-induced phenotypic adaptation can be used as a targeted strategy to enhance biomineralization efficiency in *B. licheniformis*, while maintaining functional stability under cement-relevant conditions. These findings provide a novel framework for tailoring bacterial performance in self-healing systems for construction biotechnology.

## 1. Introduction

An increasing number of concrete infrastructure around the world, which are approaching the end of their service life, demands innovative strategies for preservation and retrofitting [1]. At the same time, there is a growing need for additional infrastructure which requires the development of innovative solutions, especially when it comes to sustainable structural concrete [2]. However, cracking remains a fundamental durability issue in concrete infrastructure. Even microcracks can act as preferential pathways for water and aggressive ions, accelerating structural degradation and reinforcement corrosion. This ultimately leads to a reduction or loss of load-bearing capacity [3]. Over the past 15 years, bacteria-based self-healing has emerged as a promising bio-enabled strategy for “autonomous” durability preservation. This approach relies on CaCO_3_ precipitation within microcracks and cracks, most often mediated by alkali-resistant, spore-forming *Bacillus* strains [4]. However, performance under realistic service conditions remains highly dependent on several factors, including metabolic pathway selection, bacterial survival and activation within the cementitious matrix, and the compatibility of nutrients and metabolic by-products with durability and environmental requirements [5]. In parallel, ultraviolet (UV) exposure has been explored both as a microbial inactivation method and, at sublethal doses, as a tool for generating phenotypic mutants with enhanced biomineralization capacity. This suggests a potential “*ready-to-adapt*” strategy that could complement the conventional “*ready-to-use*” selection of natural isolates [6,7]. Concrete and other cement-based materials exhibit a limited autogenous self-healing capacity, primarily driven by continued hydration, pozzolanic reactions, and CaCO_3_ precipitation within cracks. However, this intrinsic mechanism is strongly constrained by crack width, moisture availability, and environmental conditions, and is generally insufficient to ensure long-term durability [8,9]. Bacteria-based self-healing is typically discussed under the umbrella of microbiologically induced carbonate precipitation (MICP), but MICP is not a single mechanism. Additionally, MICP studies have focused on the selection of naturally occurring strains or optimization of environmental conditions, while limited attention has been given to targeted phenotypic adaptation strategies for improving biomineralization performance under cement-relevant conditions. In particular, although UV-induced mutagenesis has been reported to enhance ureolytic activity and mineralization in some microorganisms, its potential as a controlled tool for tuning the self-healing performance of *Bacillus* strains remains insufficiently explored. Therefore, a clear knowledge gap exists in understanding whether controlled UV-induced phenotypic adaptation can be used to systematically enhance biomineralization efficiency while maintaining physiological robustness in conditions relevant to cementitious materials. A key implication for materials engineering is that different pathways can produce precipitates with different morphologies and organic/inorganic character, which can plausibly alter crack-filling integrity and durability performance [10,11]. Comparing ureolytic and non-ureolytic environmental bacteria shows that ureolytic strains can produce rapid, widespread pH changes with relatively homogeneous fine crystals, while non-ureolytic strains may form larger, mixed organic/inorganic precipitates and can deliver robust crack-healing performance despite slower kinetics [12].

Spore-forming *Bacillus*-related bacteria are natural candidates for cementitious environments because endospores can tolerate desiccation, nutrient scarcity, and other stressors, and can remain dormant until crack-driven water ingress enables germination and activity [13]. Early bacteria-based self-healing work explicitly selected alkali-resistant spore-formers and demonstrated that spores can remain viable for months after incorporation, although progressive pore refinement during cement hydration can physically constrain spore survival and activation [14,15]. *Bacillus licheniformis* aligns with this selection logic because it is a robust spore-former and can sporulate across a broad environmental envelope which supports its plausibility as a durable “bio-agent” when appropriately protected and delivered [16,17,18,19]. *B. licheniformis* is also moving from general acceptability toward application-specific evidence. Namely, recent studies using *B. licheniformis* with porous carriers report improvements in crack-related performance, including compressive strength linked to bacterial self-healing effects [17,18,20]. This supports the rationale for testing *B. licheniformis* references strains as well as isolates within advanced, engineering-targeted self-healing frameworks.

UV exposure occupies a dual role relevant to self-healing concrete as an inactivation stressor that can reduce viability, and as a bacterial strain improvement tool through sublethal mutagenesis [21,22,23]. The latter has direct precedent in biomineralization since UV mutants of ureolytic bacteria have shown increased urease activity, enhanced calcite production, and improved survival at higher pH compared with wild-type strains [24,25,26,27]. This demonstrates that UV can, under controlled regimes, shift biomineralization phenotypes in ways that are potentially advantageous for cementitious environments [28]. At the same time, *Bacillus* spores exhibit strong resistance properties that complicate simplistic UV assumptions. Spore structure and protective chemistry confer resistance to multiple physical treatments including UV, and treatment efficacy depends strongly on dose, wavelength, and environmental context [29].

The main objective is to determine whether UV treatment makes an alkaline soil *B. licheniformis* isolate more “ready-to-adapt” for civil engineering self-healing application by quantifying how UV changes survivability under extreme alkalinity, as well as biomineralization. The central hypotheses are that UV-treated *B. licheniformis* can exhibit enhanced biomineralization-related phenotypes translating into improved crack healing. The additional aim of this study was to apply artificial neural networks (ANNs) as an effective mathematical tool for modeling systems characterized by high variability and nonlinear relationships among variables [16,30], which are typical features of the self-healing potential of *B. licheniformis* natural isolates and their phenotypic mutants. In this context, ANN modeling was specifically employed to capture complex interactions among key environmental and biochemical factors, including pH, urea concentration, and salinity, that govern ureolytic activity and calcium carbonate (CaCO_3_) precipitation. Such an approach enables robust prediction of biomineralization performance based on experimental data, providing a complementary framework to conventional assays for evaluating bacterial self-healing efficiency. In recent years, ANN approaches have increasingly been employed to investigate complex biological processes, including microbiologically induced mineralization and self-healing mechanisms, due to their strong capability to predict and integrate multiple interrelated parameters influencing bacterial activity and biomineralization performance [4]. These modeling approaches are particularly valuable in industrial and biotechnological applications involving bacterial self-healing systems, where conventional experimental assays may be time- and resource-intensive. Unlike traditional mechanistic models, ANN models do not require predefined physical parameters. Instead, they rely on experimental datasets to learn underlying patterns and relationships. Consequently, ANN systems are capable of efficiently handling nonlinear processes and capturing complex interactions among variables within biological systems, thereby providing a powerful predictive framework for evaluating the self-healing potential of bacteria.

## 2. Materials and Methods

**Figure 1:**
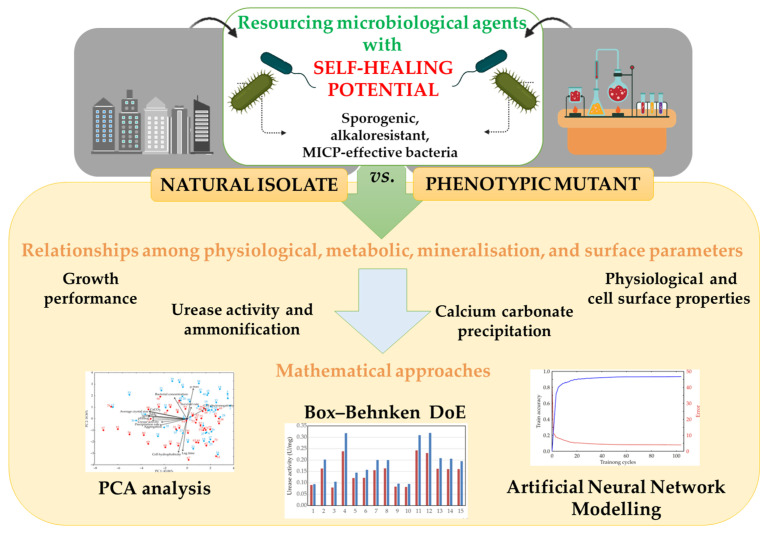
Experimental steps.

This study was designed to compare the physiological robustness and biomineralization potential of a natural alkaline soil isolate of *Bacillus licheniformis* with its UV-adapted derivative using laboratory proxies of “self-healing potential”. The experimental workflow is presented in Figure 1.

### 2.1. Bacterial Strain and Culture Conditions

A urease-positive, spore-forming strain of *Bacillus licheniformis* previously isolated from calcite-rich alkaline soil near Novi Sad, Serbia, was used in this study [31,32]. The isolate was previously characterized as a ureolytic, alkalotolerant strain with demonstrated capability for MICP, exhibiting efficient pH increase, calcium ion reduction, and stable biomineralization performance under alkaline conditions [31,32]. The strain was maintained in 25% (*v*/*v*) glycerol stocks at −80 °C. Working cultures were propagated in Nutrient Broth (NB) at 30 °C with shaking at 150 rpm. For mineralization experiments, a urea–calcium mineralization medium (UCMM) containing 20 g/L urea, 10 mM CaCl_2_·2H_2_O, 5 g/L yeast extract, and 5 g/L NaCl was used. The initial pH was adjusted to 8.5 prior to sterilization.

### 2.2. UV Irradiation and Adaptive Evolution

Exponentially growing cultures (OD600 = 0.6) were harvested by centrifugation at 6000× *g* for 10 min, washed twice with sterile phosphate-buffered saline (PBS), and resuspended to approximately 10^8^ CFU/mL Aliquots were transferred into sterile Petri dishes forming a liquid layer < 2 mm to ensure uniform UV exposure. Irradiation was performed using a calibrated UV lamp (254 nm) at a fixed distance of 20 cm. Samples were exposed to doses of 0 (control), 50, 100, 200, and 400 J/m^2^. The UV dose was calculated as radiation intensity multiplied by exposure time. Following irradiation, samples were incubated in darkness for 1 h to prevent photoreactivation. Serial dilutions were plated to determine survival rates. Colonies surviving sublethal UV doses (100–200 J/m^2^) were selected for adaptive evolution [33,34,35]. Adaptive evolution was conducted through ten sequential passages under combined stress conditions, including alkaline pH (10.0), 5% NaCl, and 40 g/L urea. This procedure generated the following:

• UV-adapted strain (UV-strain in the following text), which was compared to;
• The untreated natural strain (N-strain in the following text).

Colonies surviving sublethal UV doses were further screened based on a combination of growth and metabolic criteria. Selection was performed by evaluating: (i) visible colony formation and growth rate under alkaline and saline conditions, (ii) colony morphology indicative of stable phenotypic traits, and (iii) preliminary urease activity assessed using a rapid colorimetric urea hydrolysis test. Colonies exhibiting consistent growth and positive ureolytic response were selected for subsequent adaptive evolution cycles.

### 2.3. Growth Performance and Survival

Growth kinetics were evaluated using a microplate reader by measuring optical density at 600 nm every 30 min for 72 h. Growth parameters including maximum specific growth rate and lag phase duration were calculated using the modified Gompertz model. In parallel, viable cell counts were determined by plate counting at selected time intervals to validate optical density measurements.

### 2.4. Determination of Urease Activity

Urease activity was quantified using two complementary approaches. Qualitative assessment was performed in urea broth containing phenol red indicator, where color change from yellow to pink indicated urea hydrolysis. Quantitative urease activity was measured using the phenol-hypochlorite method. Culture supernatants were incubated with 2% urea solution at 37 °C for 30 min. Released ammonium was quantified spectrophotometrically at 630 nm. Enzyme activity was expressed as µmol NH_4_^+^ released per minute per milligram of protein. Changes in pH during ureolysis were continuously monitored using a calibrated pH electrode.

### 2.5. Precipitation Potential

Biomineralization potential was evaluated in UCMM under shaking conditions (30 °C, 150 rpm) for 72 h. At defined time points (24, 48, and 72 h), samples were collected for mineral quantification. Precipitates were harvested by centrifugation at 8000× *g* for 15 min, washed three times with distilled water, and dried at 40 °C to constant weight. Precipitation yield was expressed as mg CaCO_3_ per 100 mL of culture medium.

### 2.6. Physiological and Surface Properties

To investigate physiological differences between N- and UV-strains, cell surface properties were analyzed. Zeta potential measurements were performed using a zeta potential analyzer to determine surface charge. Bacterial suspensions were prepared in distilled water at standardized cell density. Cell surface hydrophobicity was evaluated using the microbial adhesion to hydrocarbons (MATH) assay [32]. Bacterial suspensions were mixed with hexadecane, vortexed, and allowed to separate. Hydrophobicity index was calculated as the percentage reduction in aqueous phase optical density. Auto-aggregation ability was determined by monitoring the decrease in OD600 of static cell suspensions over time. Aggregation percentage was calculated relative to initial OD.

### 2.7. Experimental Design

A Box–Behnken experimental design was employed to systematically investigate the effects of pH (8.5–10.5), urea concentration (10–50 g/L), and NaCl concentration (1–7%) on biomineralization performance. Fifteen design points, including three center points, were tested for both strains (Supplemental Table S1). Each experimental condition was conducted in triplicate, and measurements were performed at 24, 48, and 72 h. The experiment evaluated the influence of environmental factors (initial pH 8.5–10.5, urea concentration 10–50 g/L, and NaCl concentration 1–7%) on growth kinetics, ureolytic activity, mineralization capacity, and surface properties of N- and UV-strains.

### 2.8. Artificial Neural Network (ANN)

The multilayer perceptron (MLP) model, recognized for its strong capability to approximate complex nonlinear relationships, was employed. Model optimization was performed using the Broyden–Fletcher–Goldfarb–Shanno (BFGS) training algorithm with 100,000 iterative cycles. To improve the robustness and generalization capability of the model, a five-fold cross-validation procedure was additionally applied. In this approach, the dataset was randomly divided into five approximately equal subsets. During each iteration, four subsets (80% of the data) were used for model training, while the remaining subset (20%) served for validation/testing. This procedure was repeated five times so that each subset was used once as a validation set. The final model performance was obtained by averaging the results from all five folds, thereby reducing the risk of overfitting and providing a more reliable estimation of predictive capability. Within each fold, the experimental data were further organized into training (70%), cross-validation (15%), and testing (15%) subsets to monitor model convergence and prediction accuracy. The synaptic weights and bias parameters associated with the hidden and output layers were arranged into two matrices, denoted as *W*_1_ and *B*_1_ for the hidden layer and *W*_2_ and *B*_2_ for the output layer, respectively.

(1) $$Y=f_{1}(W_{2}\cdot f_{2}(W_{1}\cdot X+B_{1})+B_{2})$$

Weight coefficients were determined during the ANN learning cycle, with the intention to minimize the error between network results and experimental values [36,37].

### 2.9. Statistical Analysis

All experiments were conducted in biological triplicates unless otherwise specified. Correlation analysis and principal component analysis (PCA) were performed to identify relationships among parameters. Statistical analysis of the data, including ANN modeling and construction of the MLP architecture, was performed using the software package STATISTICA 10.0 (StatSoft Inc., Tulsa, OK, USA).

## 3. Results

Supplement Table S2 summarizes the raw data of physiological, biochemical, and mineralization-related parameters used to evaluate the self-healing potential of N- and UV-strains of *B. licheniformis* under different environmental conditions. The selected indicators include microbial growth characteristics, ureolytic activity, alkalization capacity, CaCO_3_ precipitation efficiency, and bacterial surface properties, which collectively determine the effectiveness of MICP in self-healing systems [38].

### 3.1. Screening Differences in N- and UV- B. licheniformis Strains

**Figure 2:**
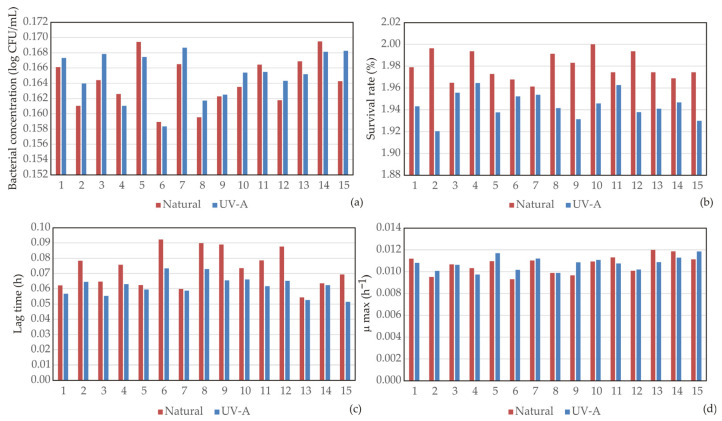
Slopes of temporal variation in key bacterial growth-related parameters of *B. licheniformis* during incubation periods of 24, 48, and 72 h: (**a**) bacterial concentration (log CFU/mL), (**b**) survival rate (%), (**c**) lag time (h), (**d**) μ_max_ (h^−1^).

**Figure 3:**
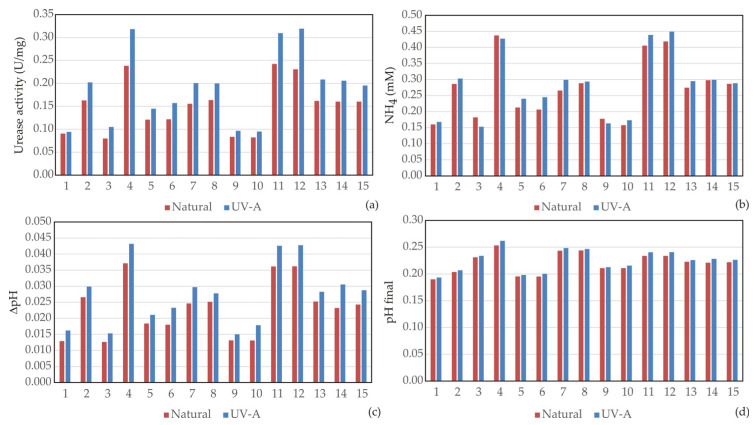
Slopes of temporal variation in ureolytic activity-related parameters of *B. licheniformis* during incubation periods of 24, 48, and 72 h: (**a**) urease activity (U/mg), (**b**) NH_4_ (mM), (**c**) ΔpH, (**d**) pH final.

**Figure 4:**
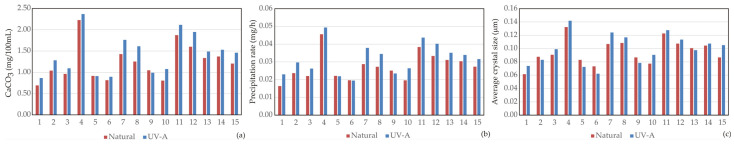
Slopes of temporal variation for precipitation-related parameters of *B. licheniformis* during incubation periods of 24, 48, and 72 h: (**a**) CaCO_3_ (mg/100 mL), (**b**) precipitation rate (mg/h), (**c**) average crystal size (μm).

**Figure 5:**
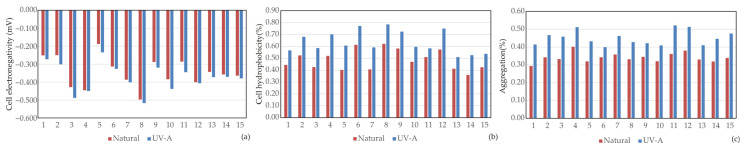
Slopes of temporal variation in cell surface-related parameters of *B. licheniformis* during incubation periods of 24, 48, and 72 h: (**a**) cell electronegativity (mV), (**b**) cell hydrophobicity (%), (**c**) aggregation (%).

Microbial growth and viability were assessed through bacterial concentration, survival rate, lag phase duration, and the maximum specific growth rate (Figure 2). These parameters provide insight into the physiological stability of the strains under alkaline and saline conditions which can be present in cementitious environments [39]. The metabolic potential for carbonate formation was evaluated by measuring urease activity, ammonium production, changes in pH, and the final pH of the medium, reflecting the efficiency of urea hydrolysis and the resulting alkalization process (Figure 3). The mineralization performance of the strains was quantified through CaCO_3_ production, precipitation rate, and average crystal size (Figure 4), which indicate the intensity of crystals formation. In addition, bacterial surface characteristics, such as cell electronegativity, hydrophobicity, and aggregation were analyzed (Figure 5), as these properties influence calcium ion adsorption, nucleation processes, and potential biofilm formation, all of which are critical for effective biomineralization and crack healing in self-healing materials [31,32]. This combination of parameters provides a comprehensive evaluation of bacterial physiological performance and mineralization capability, enabling a comparative assessment of the self-healing efficiency of the investigated strains.

#### 3.1.1. Growth Performance and Survival

Figure 2 presents the slopes of the temporal changes in key parameters for growth include bacterial concentration, survival rate, lag time, and maximum specific growth rate of both tested *B. licheniformis* for incubation periods of 24, 48, and 72 h. The slopes represent the rate of change in each measured variable over time and therefore provide insight into the dynamics of bacterial growth. Both bacterial strains exhibited relatively stable bacterial concentrations (approx. 0.16–0.17 log CFU/mL) across the tested conditions, indicating that the selected environmental factors did not substantially inhibit cell growth. Survival rates were slightly higher for the N-strain (1.96–2%) compared with the UV-strain (1.92–1.96%), suggesting marginally better physiological stability of the N-strain under alkaline and saline environments. Lag phase duration varied between 0.05 and 0.09 h for both strains, with slightly longer lag times observed at elevated NaCl concentrations (7%), indicating osmotic adaptation. The maximum specific growth rate remained nearly constant across all treatments, demonstrating that variations in environmental conditions primarily influenced metabolic activity rather than growth kinetics.

Univariate ANOVA showed that NaCl concentration significantly affected bacterial concentration (*p* < 0.001) and lag time (*p* < 0.01), while no significant effects of strain, pH, or urea were observed for bacterial concentration. Survival rate was significantly influenced by bacterial strain (*p* < 0.001), indicating strain-dependent physiological differences. Lag time was additionally significantly affected by bacterial strain (*p* < 0.001), confirming differences in adaptation dynamics between N- and UV-strains. For μ_max_, a significant effect of NaCl (*p* < 0.01) and a significant urea × NaCl interaction (*p* < 0.05) were observed, while bacterial strain was not significant.

#### 3.1.2. Urease Activity and Ammonification

As can be seen in Figure 3, urease activity strongly depended on urea concentration. Under low urea levels, both strains exhibited relatively low enzymatic activity (≈0.08–0.10 U/mg). Increasing urea concentration significantly enhanced urease activity, reaching 0.24 U/mg for the N-strain and up to 0.32 U/mg for the UV-strain. This increased enzymatic activity resulted in higher ammonium production, which ranged from approximately 0.15–0.18 mM at low urea concentrations to about 0.43–0.45 mM at high concentrations of urea. The enhanced ammonification directly contributed to alkalization of the medium, as reflected by increased ΔpH values. The UV-strain consistently exhibited slightly higher urease activity and ammonium production compared with the N-strain, suggesting that phenotypic mutation may have enhanced ureolytic expression or enzyme efficiency. The final pH values ranged from 0.19 to 0.26 (expressed as relative scale units in the dataset), with the highest values recorded at elevated initial pH (10.5) and high urea concentrations.

The regression analysis (Table 1) was performed to quantitatively model the relationships between environmental factors (pH, urea concentration, NaCl, and incubation time) and ammonium-related response as a proxy for ureolytic activity. This approach enabled the evaluation of both linear and nonlinear effects, as well as interaction terms, which are not fully captured by univariate comparisons. The regression results for ammonium-related response (as a proxy for ureolytic activity) indicate strong model fits for both datasets (*R*^2^ = 0.965 for N-strain; *R*^2^ = 0.967 for UV-strain), confirming the robustness of the predictive models. Importantly, urea concentration was identified as a statistically significant predictor of ammonium-related response in both strains, with a consistently stronger effect observed in the UV-strain, as reflected by the larger magnitude of the linear urea coefficient. In contrast, NaCl showed no significant main effect, while several significant interaction terms, particularly involving pH and urea, highlight the coupled regulation of nitrogen metabolism. These regression outputs enable direct estimation of absolute ammonium-related responses across the experimental domain, improving interpretability beyond slope-based comparisons. The models confirm that the UV-strain exhibits consistently higher predicted ammonium production at elevated urea concentrations, supporting its enhanced ureolytic activity and biomineralization potential.

The obtained results indicate that urease-mediated hydrolysis effectively increased alkalinity in the system and promoted carbonate supersaturation. Both strains demonstrated similar alkalization capacity, but the UV-strain produced slightly higher ΔpH values in several treatments, consistent with its increased urease activity.

#### 3.1.3. CaCO_3_ Precipitation

CaCO_3_ precipitation showed strong dependence on both urea concentration and initial pH value (Figure 4). At low concentrations of urea, CaCO_3_ production remained relatively low (0.69–1.10 mg/100 mL). In contrast, increasing the urea concentration resulted in a substantial increase in mineralization efficiency.

The highest CaCO_3_ production was observed at pH 10.5 and 50 g/L urea, reaching 2.23 mg/100 mL for the N-strain and 2.37 mg/100 mL for the UV-strain. These conditions also corresponded to the highest precipitation rates (approx. 0.05 mg/h). Moderate NaCl concentrations (1–4%) generally favored precipitation, whereas high salinity (7%) slightly reduced CaCO_3_ yield, indicating partial osmotic inhibition of mineralization processes. Crystal size increased with increasing mineralization intensity. The UV-strain produced slightly larger crystals (up to 0.14 μm) compared with the N-strain (approx. 0.13 μm) which suggests that enhanced urease activity may accelerate nucleation and crystal growth.

#### 3.1.4. Physiological and Cell Surface Properties

Surface characteristics differed noticeably between the strains (Figure 5). Both strains displayed negative surface charges (−0.19 to −0.52 mV), which favors the attraction of Ca^2+^ ions and supports nucleation of CaCO_3_ crystals. The slightly more negative values observed at higher pH may further enhance ion binding and mineral precipitation (Figure 5a). The UV-strain exhibited higher cell hydrophobicity compared with the N-strain (Figure 5b). Similarly, aggregation levels were higher in the UV-strain than in the N-strain (Figure 5c).

### 3.2. Evaluate the Relationships Among Physiological, Biochemical, Mineralization, and Surface Parameters of N- and UV-Bacillus licheniformis

**Figure 6:**
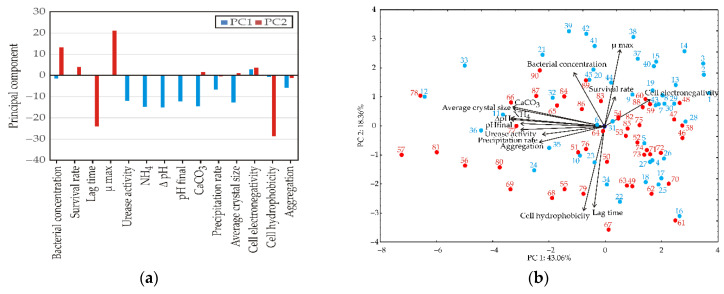
(**a**) PCA score plot showing separation of biomineralization efficiency (PC1) and physiological/surface traits (PC2) in *B. licheniformis* strains; (**b**) PCA biplot of physiological, biomineralization, and cell surface parameters influencing the self-healing potential of *B. licheniformis* strains. Blue dots represent N-strain, whereas red dots correspond to UV-strain. The identification numbers of all samples are listed in Supplementary Material Table S2.

Principal component analysis was applied to evaluate the relationships among physiological, biochemical, mineralization, and surface parameters of *Bacillus licheniformis* and to identify the dominant factors influencing the self-healing potential (Figure 6). The first two principal components explained 56.27% of the total variance in the dataset. As can be seen in Figure 6a, the first principal component (PC1) had an eigenvalue of 5.66 and accounted for 40.42% of the total variability, while the second principal component (PC2) had an eigenvalue of 2.22, explaining an additional 15.85% of the variance. Additionally, Figure 6b indicates that the first principal component (PC1) was primarily associated with variables related to ureolysis, alkalization, and CaCO_3_ precipitation. Strong negative loadings were observed for ΔpH (−15.18%, based on correlation), NH_4_^+^ (−14.91%), CaCO_3_ production (−14.64%), average crystal size (−12.73%), pH_final (−12.28%), urease activity (−11.98%), and precipitation rate (−6.76%). These parameters are directly linked to MICP and collectively describe the intensity of biomineralization processes [31]. The strong correlation among urease activity, ammonium production, pH increase, and CaCO_3_ formation indicates that ureolytic metabolism represents the dominant mechanism controlling mineral precipitation. As urease hydrolyzes urea, ammonium and carbonate ions are produced, resulting in alkalization and subsequent CaCO_3_ formation. Consequently, PC1 can be interpreted as the biomineralization efficiency component, integrating metabolic activity and mineral precipitation capacity.

The second principal component (PC2) was mainly influenced by cell surface properties and growth kinetics. The highest loadings were observed for cell hydrophobicity (−28.63% based on correlation), lag time (−24.11%), µ_max_ (21.15%), and bacterial concentration (13.27%). These variables describe physiological adaptation and cell surface behavior, which affect microbial adhesion, aggregation, and nucleation processes. High hydrophobicity and aggregation can promote the formation of nucleation sites for CaCO_3_ precipitation, while growth parameters determine the speed at which bacterial populations establish metabolic activity. Therefore, PC2 reflects physiological and surface-related factors influencing nucleation and early-stage mineral formation rather than the overall amount of precipitated CaCO_3_.

### 3.3. ANN Modeling

#### 3.3.1. ANN Model

**Figure 7:**
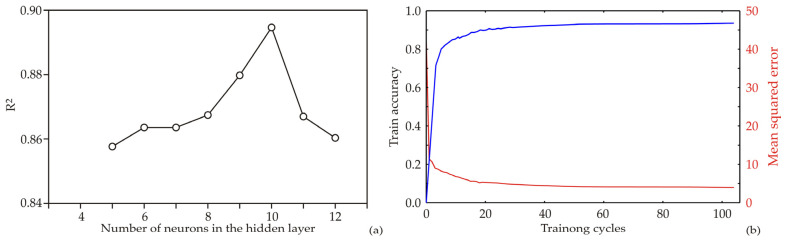
ANN calculation: (**a**) The dependence of the *R*^2^ value of the number of neurons in the hidden layer in the ANN model, (**b**) training results per epoch.

The architecture of the ANN, including bias terms and weight coefficients, strongly depends on the initial assumptions regarding matrix parameters, which are crucial for proper model construction and fitting to experimental data. Furthermore, the number of neurons in the hidden layer significantly influences the predictive performance and stability of the ANN model. To minimize the effect of random initialization and potential spurious correlations, each network topology was executed 100,000 times, thereby ensuring stable convergence and reliable parameter estimation. Following this optimization procedure, the highest coefficient of determination (*R*^2^) during the training process was achieved when nine neurons were used in the hidden layer, which was therefore selected as the optimal ANN configuration (Figure 7a). The selected model was subsequently trained for 100 epochs, and the evolution of the training accuracy and training loss during the learning process is illustrated in Figure 7b.

The training accuracy increased progressively with the number of training cycles until approximately the 60th–70th epoch, where it approached a plateau. At this stage, the highest training accuracy and lowest loss values were observed. Beyond this point, only marginal improvements in accuracy and slight decreases in loss were detected, indicating the onset of overfitting. Consequently, extending the training beyond 80 epochs could lead to excessive overfitting, whereas approximately 60 epochs appear sufficient to achieve high predictive accuracy while maintaining good model generalization capability (Figure 7b).

#### 3.3.2. Performance of the Optimal ANN Model

The optimal ANN model (Table 2) identified in this study was the multilayer perceptron MLP 6-10-14, which consists of six input neurons, ten neurons in the hidden layer, and fourteen output neurons. This architecture was selected as the best-performing model based on the fivefold cross-validation procedure. The model achieved training, testing, and validation performance coefficients of 0.802, 0.765, and 0.730, respectively, indicating a relatively strong agreement between predicted and experimental values across all data subsets. The small difference between these performance values suggests that the model demonstrates good generalization ability, without significant overfitting to the training data. Error analysis further supports the reliability of the developed ANN model. The training error (46.665) was lower than the testing error (59.387) and validation error (61.864), which is expected since the model parameters are optimized using the training dataset. However, the moderate increase in errors for the testing and validation datasets indicates that the model maintains acceptable predictive accuracy when applied to unseen data. The network was trained using the Broyden–Fletcher–Goldfarb–Shanno (BFGS) optimization algorithm, which is a quasi-Newton method known for efficient convergence in nonlinear optimization problems. The sum-of-squares (SOS) error function was applied during the training process to minimize the difference between predicted and observed values. Both the hidden and output layers employed logistic activation functions, enabling the model to capture nonlinear relationships between the input variables and the predicted responses. Such nonlinear modeling capability is particularly important for complex biological and physicochemical processes [40], including the metabolic activity and biomineralization behavior of *B. licheniformis*. The obtained results indicate that the MLP 6-10-14 architecture provides a stable and reliable predictive model, capable of describing the relationships between environmental factors and multiple physiological and biomineralization parameters simultaneously. The balanced performance across training, testing, and validation datasets confirms that the developed ANN model can be effectively used for prediction and optimization of bacterial self-healing processes under varying environmental conditions.

**Figure 8:**
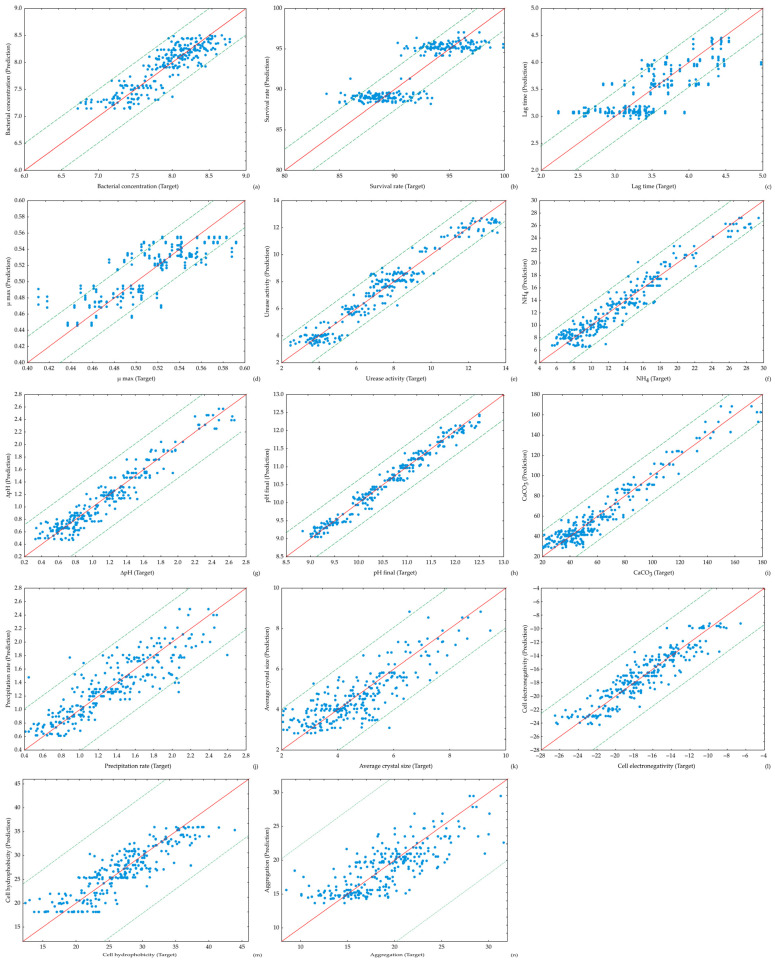
Target-prediction plots illustrating the agreement between measured and predicted values of physiological, biochemical, and mineralization-related parameters of *B. licheniformis* during incubation periods of 24, 48, and 72 h: (**a**) bacterial concentration, (**b**) survival rate, (**c**) lag time, (**d**) μ_max_, (**e**) urease activity, (**f**) NH_4_, (**g**) ΔpH, (**h**) pH final, (**i**) CaCO_3_, (**j**) precipitation rate, (**k**) average crystal size, (**l**) cell electronegativity, (**m**) cell hydrophobicity, (**n**) aggregation.

The ANN models demonstrated a high capability to accurately predict the experimental variables across a broad range of process conditions. This is illustrated in Figure 8, which presents the comparison between the experimentally measured values and those predicted by the ANN model, indicating a strong agreement between the two datasets.

#### 3.3.3. Predictive Performance of the ANN Model Based on R^2^ Values

The predictive capability of the optimal ANN model MLP 6-10-14 (Table 2) was evaluated using *R*^2^ for the training, testing, and validation datasets. These results provide insight into the ability of the model to accurately reproduce the physiological, biochemical, mineralization, and surface characteristics of *B. licheniformis*. The ANN model demonstrated high predictive accuracy for several key metabolic and biomineralization parameters, while moderate predictive performance was observed for variables associated with bacterial growth dynamics and cell surface properties.

The highest *R*^2^ values were obtained for pH final, which showed excellent predictive accuracy across all datasets (0.976 for training, 0.983 for testing, and 0.971 for validation). Similarly, urease activity, NH_4_^+^ concentration, ΔpH, and CaCO_3_ production exhibited strong correlations between experimental and predicted values, with *R*^2^ values generally above 0.90 in the training and testing phases and remaining above 0.85 during validation cycle. These results indicate that the ANN model successfully captured the relationships governing ureolytic metabolism and subsequent biomineralization processes, which are responsible for MICP. Because these variables are closely mechanistically linked [38], their high predictability reflects the ability of the ANN to model these interconnected biochemical pathways. Moderate predictive performance was observed for bacterial concentration, survival rate, precipitation rate, average crystal size, cell electronegativity, and cell hydrophobicity, with *R*^2^ values generally ranging from 0.58 to 0.89 across the datasets. The consistency of these values between training, testing, and validation sets indicates that the model maintains reasonable stability when predicting these parameters. Among these variables, cell electronegativity exhibited relatively strong predictive performance (up to 0.894 in the testing set). This suggests that the ANN captured the main environmental influences affecting bacterial surface charge. The lowest *R*^2^ values were obtained for lag time, µ_max_, and aggregation, where values ranged approximately between 0.56 and 0.66 across the datasets. These lower coefficients suggest that these parameters are influenced by more complex biological variability or stochastic cellular processes that are more difficult to describe using the selected input variables. Future improvements of the model may therefore benefit from the integration of additional biological descriptors, such as transcriptomic profiles, EPS composition, and microenvironmental heterogeneity, which are known to influence cell–cell interactions and mineralization behavior. In particular, aggregation behavior and growth kinetics may depend on additional factors such as microenvironmental interactions, cell–cell communication, or experimental variability, which were not fully captured in the model. Importantly, the *R*^2^ values for the training, testing, and validation datasets were generally similar, indicating that the ANN model did not suffer from significant overfitting and maintained good generalization ability. The relatively stable predictive performance across datasets confirms that the MLP 6-10-14 architecture provides a robust tool for predicting biomineralization-related parameters.

#### 3.3.4. Verification of the ANN Model

Table 3 presents the statistical indicators used to evaluate the predictive performance of the ANN model for physiological, biochemical, mineralization, and cell surface parameters of *B. licheniformis*. The model performance was assessed using several statistical metrics, including the chi-square statistic (χ^2^), root mean square error (RMSE), mean bias error (MBE), mean percentage error (MPE), sum of squared error (SSE), average absolute relative deviation (AARD), coefficient of determination (*r*^2^), and distribution descriptors such as skewness and kurtosis. The ANN model demonstrated good predictive capability for most of the investigated parameters, particularly those associated with metabolic activity and biomineralization processes. The highest predictive accuracy was achieved for pfinal H (*r*^2^ = 0.976), indicating an excellent agreement between experimental and predicted values. Similarly, urease activity (*r*^2^ = 0.943), NH_4_^+^ concentration (*r*^2^ = 0.928), ΔpH (*r*^2^ = 0.928), and CaCO_3_ production (*r*^2^ = 0.929) exhibited strong correlations, suggesting that the ANN model effectively captured the relationships governing ureolysis-driven biomineralization.

The relatively low RMSE values observed for these parameters further support the reliability of the model predictions. For instance, RMSE values of 0.704 for urease activity, 1.444 for NH_4_^+^, and 0.150 for pH final indicate limited deviation between predicted and measured data. Moreover, the mean bias error (MBE) values were close to zero for most variables, implying the absence of systematic overestimation or underestimation by the model. Moderate predictive performance was observed for bacterial concentration (r^2^ = 0.774), precipitation rate (*r*^2^ = 0.756), and cell hydrophobicity (*r*^2^ = 0.772), indicating that these parameters were partially influenced by complex interactions that may not have been fully captured by the model structure. Lower coefficients of determination were obtained for lag time (*r*^2^ = 0.577), µ_max_ (*r*^2^ = 0.620), aggregation (*r*^2^ = 0.608), and average crystal size (*r*^2^ = 0.698), suggesting greater variability and reduced model predictability for parameters related to bacterial growth dynamics and microstructural characteristics of precipitated minerals. The distribution analysis of residuals showed skewness values close to zero and kurtosis values near the normal distribution range, indicating that the prediction errors were approximately normally distributed. This confirms that the ANN model did not produce strongly biased predictions and that the residual errors were relatively symmetrical. Although higher SSE values were observed for parameters with larger absolute magnitudes, such as CaCO_3_ production, cell hydrophobicity, and aggregation, this is expected due to the scale of these variables rather than model inadequacy. In general, the ANN model successfully reproduced the experimental trends, particularly for parameters associated with ureolytic metabolism and CaCO_3_ precipitation, which represent the key mechanisms responsible for biomineralization and self-healing performance by *B. licheniformis*.

The skewness and kurtosis values across all ANN models indicate generally well-behaved and approximately normal residual distributions. Specifically, skewness values ranged from −0.351 (µ_max_) to 0.202 (cell hydrophobicity), with most responses clustering very close to zero (bacterial concentration = 0.099; NH_4_ = 0.096; CaCO_3_ = 0.048), indicating a high degree of symmetry in prediction errors. Similarly, kurtosis values were mostly close to zero, varying from −0.211 (CaCO_3_) and −0.195 (lag time) to 0.606 (aggregation) and 1.911 (precipitation rate), with the majority of variables showing mild or negligible deviation from normality. Only minor departures from normality were observed, with slightly higher kurtosis for precipitation rate (1.911) and aggregation (0.606), which may reflect increased heterogeneity in these biologically more variable responses. The combination of low skewness and generally low kurtosis supports the conclusion that residuals are approximately normally distributed, indicating robust ANN model fitting and absence of systematic prediction bias. The results indicate that the developed ANN model can serve as a reliable predictive tool for evaluating and optimizing bacterial self-healing systems under varying environmental conditions.

## 4. Discussion

The screening experiment discovered a coherent relationship between bacterial physiology, ureolytic metabolism, and mineralization performance under alkaline and saline conditions. Both strains followed the expected progression of ureolysis-driven biomineralization, where urea hydrolysis promotes alkalization and increases carbonate availability, ultimately enabling CaCO_3_ precipitation. However, the UV-phenotypic mutant generally exhibited higher functional outputs, particularly in urease activity and CaCO_3_ formation, whereas the N-strain demonstrated slightly higher survival stability. These trends suggest that UV-induced phenotypic modification may enhance metabolic activity without substantially compromising physiological robustness.

### 4.1. Screening Differences in N- and UV- Phenotypic B. licheniformis

The ability of bacteria to remain viable and metabolically active under highly alkaline and saline conditions represents a fundamental prerequisite for their application in cement-based self-healing systems [10]. Both the N- and the UV- *B. licheniformis* maintained relatively stable bacterial concentrations and comparable maximum specific growth rates across the tested environmental conditions. This indicates that neither moderate salinity nor elevated alkalinity substantially inhibited bacterial growth. Based on this data, it can be suggested that both strains possess physiological characteristics compatible with the extreme conditions typically encountered in the cementitious matrix such as high alkalinity and low water availability [41]. The observed stability of bacterial concentration and μ_max_ under alkaline conditions is consistent with previous studies reporting that spore-forming *Bacillus* species exhibit high tolerance to alkaline environments due to robust cell wall structures, efficient proton transport systems, and the ability to regulate intracellular pH value [42,43,44]. For example, studies investigating MICP bacteria *Sporosarcina pasteurii* and *Bacillus subtilis* demonstrated that these bioagents can maintain metabolic activity at pH values above 10, which is essential for microbial survival in cement pore solutions [45,46]. Similar alkaline tolerance has also been reported for *B. licheniformis*, where adaptive metabolic responses allow growth in environments characterized by high ionic strength and elevated pH value [47,48]. In contrast to genetic engineering approaches, which rely on targeted modification of specific genes or metabolic pathways, the UV-induced phenotypic adaptation applied in this study represents a non-specific, selection-driven strategy that exploits natural stress response mechanisms without direct genetic manipulation. This approach may offer practical advantages in terms of simplicity, lower cost, and reduced regulatory constraints, particularly for applications in construction materials where the use of genetically modified organisms may be limited.

Although the overall growth behavior of the two strains was comparable, slight differences were observed in survival rate and lag phase duration. The N-strain exhibited marginally higher survival rates, whereas the mutant showed slightly shorter lag phases under several tested conditions. These differences may reflect physiological trade-offs associated with UV-induced mutagenesis. UV exposure is known to generate random genomic modifications that can alter metabolic regulation and stressor–response pathways [6]. In some cases, such mutations enhance metabolic activity but may simultaneously reduce cellular stability under extreme environmental conditions. The slightly reduced survival rate observed in the UV mutant could therefore be associated with minor physiological costs linked to enhanced metabolic capacity. Nevertheless, the differences between strains remained relatively small, suggesting that the UV adaptation procedure did not significantly compromise bacterial viability. This observation is important from an engineering perspective because microbial self-healing agents must retain sufficient robustness to survive incorporation into the cementitious matrix and remain active after crack formation and water ingress [49]. The relatively short lag phases observed for both strains further support their suitability for self-healing applications. Rapid metabolic activation following exposure to favorable conditions, such as crack-induced water ingress, is considered critical for efficient MICP-based crack healing [50]. Lag phases below approximately 0.1 h indicate that both strains can quickly resume metabolic activity once environmental constraints are relieved, enabling timely initiation of carbonate precipitation. The growth kinetics results suggest that both N- and UV-strains of *B. licheniformis* exhibit physiological characteristics compatible with the highly alkaline and saline conditions typical of cementitious environments. The N-strain showed slightly higher survival stability, but the UV-strain maintained comparable growth performance with modestly faster adaptation dynamics.

Urease activity represents a key regulatory factor in MICP, as the hydrolysis of urea generates carbonate species and ammonia, thereby increasing local alkalinity and promoting CaCO_3_ supersaturation. In the present study, both urease activity and ammonium production increased markedly with rising urea concentration, indicating that substrate availability strongly controlled metabolic turnover. At the highest tested urea concentration (50 g/L), the UV-strain reached a urease activity of approximately 0.32 U/mg compared with 0.24 U/mg for the N-strain, corresponding to an increase of roughly one third. This higher enzymatic activity was accompanied by slightly greater ammonium production and a small increase in alkalization of the medium, reflecting more intensive ureolytic metabolism. Similar relationships between urease activity, carbonate generation, and CaCO_3_ precipitation have been widely reported for ureolytic bacteria used in MICP processes. Recent studies indicate that urease activity and biomass concentration together determine the rate of urea degradation and therefore the rate of carbonate supply in biomineralization systems. Increased enzymatic activity typically accelerates precipitation kinetics, although excessively high urease activity may also reduce spatial uniformity of mineral deposition in porous media due to rapid supersaturation near the microbial cells [51,52]. From the perspective of crack healing in cementitious materials, this kinetic advantage may be beneficial because faster carbonate generation can shorten the time required for crack healing after water ingress. At the same time, the potential trade-off between precipitation rate and spatial distribution must be considered when evaluating the practical performance of highly ureolytic strains. Another relevant aspect of ureolysis-based MICP is the formation of ammonium as a metabolic by-product. The stoichiometry of urea hydrolysis inevitably produces ammonia and ammonium ions, which may accumulate in engineered systems and represent a potential environmental concern. Recent studies have therefore explored strategies for ammonium management or alternative biomineralization pathways to reduce nitrogen emissions [53,54]. In this context, the enhanced ureolytic activity observed in the mutant should be interpreted as a kinetic advantage that may require complementary strategies for ammonium control during large-scale application.

The CaCO_3_ precipitation data followed trends similar to those observed for urease activity which confirm that carbonate supply from ureolysis strongly influenced mineralization intensity. Higher urea concentrations and elevated initial pH values resulted in increased CaCO_3_ production and precipitation rates, while moderate salinity levels were generally permissive for mineral formation. Under highly favorable conditions, the UV-strain produced slightly more CaCO_3_ than N-strain, with yields reaching approximately 2.37 mg/100 mL compared with 2.23 mg/100 mL for the N-strain. Although the difference was relatively modest, it indicates that the enhanced ureolytic metabolism of the mutant translated into increased mineralization capacity. However, total CaCO_3_ yield alone does not fully determine the engineering performance of biomineralization processes. Previous studies on MICP in porous media have demonstrated that the spatial distribution and adhesion of precipitates often play a more important role than the absolute quantity of mineral formed. For example, similar CaCO_3_ contents can produce markedly different permeability or strength responses depending on how uniformly the mineral is distributed within the pore network [55]. In the context of crack healing, well-anchored mineral bridges between crack faces may therefore be more effective than larger quantities of loosely deposited precipitate. Salinity also influenced precipitation behavior in the present study. While moderate NaCl concentrations were tolerated by both strains, the highest tested salinity slightly reduced mineralization efficiency. This observation is consistent with recent research showing that seawater ion mixtures can reduce urea decomposition rates and CaCO_3_ precipitation kinetics by lowering the effective supersaturation of carbonate minerals [56]. At the same time, ionic strength may enhance bacterial attachment to mineral surfaces by reducing electrostatic repulsion, which could influence nucleation density and precipitation localization. The average crystal size index showed a modest increase in the UV-strain compared to the N-strain. Although higher urease activity is often associated with increased nucleation density and therefore smaller crystals, crystal size in biogenic systems is influenced by several additional processes. In particular, extracellular polymeric substance (EPS) and biofilm matrix can promote aggregation of nanoscale carbonate particles and stabilize metastable mineral phases. Consequently, effective crystal size may increase through particle coalescence even when nucleation rates remain high. Recent nanoscale investigations of bacterially induced mineralization have demonstrated that EPS-rich environments facilitate the aggregation of small carbonate particles into larger mineral clusters and influence polymorph selection during crystallization [57]. The slightly larger crystal sizes observed in the mutant may therefore result from the combined effects of enhanced carbonate production and increased cell clustering or biofilm development. The differences in average crystal size observed between the strains were relatively modest. Smaller crystals may be advantageous for the early penetration and filling of fine microcracks, whereas larger crystals may contribute more effectively to bridging and sealing wider crack spaces once nucleation has been established. In this sense, crystal size alone is unlikely to determine healing efficiency. The overall crack-filling performance probably depends on the combined effects of crystal size, precipitation rate, spatial distribution, and adhesion of the mineral phase to the crack surface. Without direct crack sealing or mechanical recovery experiments and durability tests, it cannot be concluded whether the slightly larger crystal size observed for the UV-strain would improve or reduce crack-filling efficiency in real cementitious systems.

Among all measured parameters, the differences in bacterial surface properties between the two strains were particularly pronounced. The mutant exhibited substantially higher hydrophobicity and aggregation capacity compared with the N-strain, while both strains retained negative surface charge values. These characteristics are highly relevant for biomineralization processes because negatively charged bacterial surfaces and extracellular polymers can attract calcium ions and create localized zones of supersaturation around microbial cells. Electrostatic attraction between negatively charged cell surfaces and Ca^2+^ ions is widely recognized as a key step in microbial CaCO_3_ nucleation. Components such as teichoic acids, surface proteins, and extracellular polymeric substances can concentrate calcium ions at the cell interface, facilitating mineral formation once carbonate becomes available [54]. Increased cell aggregation may further amplify this effect by generating microenvironments with higher local cell density and enhanced carbonate production. In addition, the increased hydrophobicity of the mutant may promote stronger biofilm formation and attachment to solid surfaces. Biofilms and the EPS matrix are now considered important determinants of mineral morphology and deposition patterns in MICP systems. They can act as structural scaffolds that stabilize mineral phases and anchor precipitates to substrates, thereby improving resistance to detachment or washout [58]. Recent work has also shown that hydrophobic biofilm proteins can influence CaCO_3_ crystallization pathways and generate structured mineral morphologies even when bulk chemical conditions remain similar [59]. Higher hydrophobicity and aggregation in the mutant likely promote the formation of nucleation sites and facilitate biofilm-mediated mineral deposition, which may contribute to improved crack-filling capacity in self-healing materials [60,61]. The increased hydrophobicity and aggregation capacity of the UV-strain may also have direct engineering implications for self-healing performance in cementitious materials. Namely, enhanced surface adhesion and biofilm formation can improve bacterial retention within crack networks, reducing the likelihood of cell washout during water ingress. This is particularly relevant in dynamic environments, where fluid flow may otherwise limit the effectiveness of microbial self-healing agents. In addition, stronger attachment to crack surfaces may promote localized and stable mineral deposition, facilitating the formation of well-anchored CaCO_3_ bridges between crack faces.

Both strains demonstrated strong ureolytic activity and mineralization potential under alkaline conditions typical of cementitious environments. The N-strain exhibited slightly greater physiological stability and consistent mineralization across varying environmental conditions. In contrast, the UV-strain showed enhanced urease activity, increased ammonium production, higher hydrophobicity, and greater aggregation, all of which contribute to more intensive CaCO_3_ precipitation. Consequently, the UV-strain displayed the highest mineralization capacity under optimal conditions (high urea and alkaline pH), suggesting that phenotypic modification via UV exposure may enhance the self-healing efficiency of *B. licheniformis* by promoting faster and more intensive MICP. The observed trade-off between enhanced metabolic intensity and slightly reduced survival stability highlights an important design consideration for microbial self-healing systems. Strains with higher ureolytic activity, such as the UV-adapted variant, may enable faster crack sealing due to more rapid carbonate generation, which is advantageous in environments with intermittent water availability. In contrast, strains with greater physiological stability, such as the natural isolate, may ensure longer persistence within the cement matrix and more reliable activation over extended time periods. Therefore, the selection of an optimal strain is likely to depend on the specific service conditions, and in some cases, a balanced or combined strategy (targeting both metabolic performance and long-term viability) may provide the most effective approach for durable self-healing applications.

The results indicate that biomineralization efficiency arises from the interaction of metabolic activity, mineral precipitation kinetics, and microbial surface properties rather than from any single parameter alone. In the present study, the mutant combined higher urease activity with increased hydrophobicity and aggregation capacity, creating conditions that may favor rapid carbonate production and localized mineral nucleation within microbial clusters. Conversely, the N-strain demonstrated slightly higher survival stability but somewhat lower functional outputs. These findings suggest that UV-induced phenotypic adaptation can modify the balance between physiological robustness and mineralization performance. In practical applications such as bacterial self-healing concrete, the optimal strain may therefore depend on the specific operational context. Strains with higher ureolytic activity may enable faster crack healing after water ingress, whereas strains with greater survival stability may provide longer persistence within the cement matrix. The enhanced surface adhesion and aggregation behavior observed in the UV-strain may also improve retention within cracks and promote more stable mineral bridging, which could ultimately contribute to improved crack healing efficiency. Overall, the results support the hypothesis that controlled phenotypic modification can enhance functional traits relevant to biomineralization without severely compromising bacterial viability. Such strategies may therefore represent a promising approach for improving the performance of microbial self-healing systems in construction biotechnology.

### 4.2. Evaluation of the Relationships Among Physiological, Biochemical, Mineralization, and Surface Parameters of N- and UV-Strains of B. licheniformis

The PCA results indicate that the self-healing potential of the tested *B. licheniformis* strains is governed by two partially independent but biologically connected domains: a biomineralization efficiency axis represented by PC1 and a physiological/surface axis represented by PC2. Together, the first two components explained 56.27% of the total variance, which is sufficient to reveal the dominant structure of the dataset while also indicating that the system remains multifactorial, as expected for MICP-driven processes in which metabolism, solution chemistry, and cell surface behavior act simultaneously. PC1 was dominated by urease activity, NH_4_^+^ production, ΔpH, final pH, CaCO_3_ yield, precipitation rate, and average crystal size. This clustering is mechanistically consistent with the established ureolytic pathway, where urea hydrolysis drives ammonium release and alkalization, thereby increasing carbonate availability and promoting CaCO_3_ supersaturation. Recent syntheses of ureolytic MICP describe exactly this coupling: urease activity governs the rate of urea conversion, ammonium production reflects hydrolysis intensity, and pH rise shifts carbonate equilibria toward conditions favorable for mineral precipitation [51,62]. In that context, the strong co-alignment of urease, NH_4_^+^, pH-related variables, and CaCO_3_ formation in the obtained PCA supports the interpretation that ureolytic metabolism is the central driver of mineralization performance in this experimental space, rather than a secondary or strain-specific effect. This pattern also helps explain why PC1 can be interpreted as a biomineralization efficiency component rather than simply a metabolic axis. In MICP systems, higher urease activity does not act in isolation. It changes the chemical microenvironment around cells and EPS which accelerate the transition from dissolved species to precipitated mineral. That integrated behavior has been emphasized in recent work showing that urease activity controls precipitation kinetics, while carbonate supply and local alkalization jointly determine the extent and speed of CaCO_3_ formation [51,63].

At the same time, the PCA suggests that mineral formation in the system was not explained solely by carbonate supply, because PC2 captured a second layer of variation related to cell hydrophobicity, lag time, μ_max_, and bacterial concentration. This is important, because it separates how much mineral can be produced from how cells are physiologically prepared to initiate and spatially organize that production. The high contribution of hydrophobicity to PC2 indicates that cell surface properties were major discriminators among observations, likely affecting early attachment, clustering, and nucleation behavior. Contemporary studies increasingly recognize that bacterial surfaces and extracellular polymers are not passive backgrounds in MICP, but active interfaces that influence calcium binding, nucleation density, and crystal organization. EPS and biofilm-associated structures can stabilize early mineral phases, aggregate nanocrystals, and modify the morphology of the resulting carbonate deposits [64,65]. The position of lag time within PC2 is also biologically informative. A longer lag phase generally reflects greater physiological adjustment before active metabolism begins, whereas shorter lag periods indicate faster activation after exposure to favorable conditions. In the context of self-healing materials, this distinction matters because mineralization is only useful when cells can respond quickly after the formation of a microcrack and further its wetting or nutrient release. Therefore, the grouping of lag time with hydrophobicity and growth parameters suggests that PC2 describes a readiness-for-activation and surface-interaction component, which may shape the onset and localization of mineral deposition rather than total CaCO_3_ output. The PCA therefore supports a two-level interpretation of self-healing potential. First, PC1 reflects the intensity of the ureolysis–alkalization–precipitation cascade, which determines the chemical capacity of the strain to generate minerals. Second, PC2 reflects the physiological and interfacial behavior of the cells, which may control how efficiently that mineralization is initiated and spatially organized. This distinction is highly relevant for engineering applications. Previous studies have shown that similar quantities of CaCO_3_ do not necessarily produce equivalent healing outcomes, because deposition pattern, crystal adhesion, and localization within cracks or pores can be as important as total mineral content [63,66]. In other words, the PCA implies that the best-performing self-healing strain is not simply the one with the highest urease activity, but the one that combines strong metabolic output with favorable surface and growth traits. This two-axis framework may serve as a useful basis for strain selection criteria in engineered self-healing systems. Strains exhibiting high metabolic intensity (PC1) should be prioritized for rapid carbonate generation, while those with favorable physiological and surface characteristics (PC2), such as enhanced hydrophobicity and aggregation, may be better suited for efficient nucleation, adhesion, and retention within crack environments. Therefore, optimal candidates are expected to balance both dimensions, rather than maximizing a single trait, highlighting the importance of multi-parameter screening approaches in the selection and optimization of bacterial agents for construction biotechnology applications.

Another important implication of the PCA is that surface-related parameters were not tightly overlaid on the main mineralization variables, even though they are mechanistically linked. This partial separation suggests that cell surface behavior contributes indirectly to MICP performance, most likely through nucleation site formation, aggregation, and mineral anchoring rather than through direct control of carbonate chemistry. Such decoupling has also been discussed in the recent literature, where authors note that urease activity largely controls precipitation kinetics, while EPS composition, attachment behavior, and hydrophobic interfaces influence the structure and persistence of the mineral phase. This interpretation fits the gained dataset well. Namely, ureolysis-related variables define the dominant axis of variance, but surface properties still contribute substantially to differentiation among treatments and therefore cannot be neglected in strain evaluation. The PCA strengthens the broader conclusion of this study that the phenotypic mutant may offer an advantage because of enhanced ureolytic performance and altered surface behavior. If the mutant shifts toward the region associated with stronger hydrophobicity and aggregation while still aligning with the ureolysis-driven mineralization axis, then its potential benefit may lie in combining rapid carbonate generation with more favorable nucleation and retention behavior. This would be especially relevant in crack-healing scenarios, where localized mineral deposition and adhesion to crack walls are critical for durable healing. Recent reviews of microbial self-healing materials emphasize that future strain selection should move beyond simple measurements of urease activity and include traits in terms of the environmental stress adaptation, attachment, and biofilm-assisted mineralization [62,64]. The PCA confirms that the self-healing potential of *B. licheniformis* cannot be reduced to a single trait. Instead, it emerges from the interaction of metabolic intensity, chemical alkalization, CaCO_3_ precipitation, and cell surface behavior. In the formed system, PC1 captures the core biomineralization machinery, whereas PC2 captures the physiological and interfacial features that likely determine how efficiently that machinery is translated into practical healing performance. This multidimensional structure provides a strong rationale for evaluating both natural isolates and adapted phenotypic variants through integrated screening rather than by single-parameter assays alone [18,66].

### 4.3. ANN Modeling

ANN analysis discovered that the relationships between environmental factors and the physiological and biomineralization responses of *B. licheniformis* are inherently nonlinear and interconnected. Microbial biomineralization processes such as MICP are influenced simultaneously by metabolic activity, solution chemistry, and microbial surface properties. Because these mechanisms interact dynamically, conventional linear statistical approaches often fail to adequately capture the complexity of such systems. In contrast, machine learning models, particularly multilayer perceptrons, can approximate nonlinear relationships and detect hidden patterns in multidimensional datasets [32,67]. The optimization procedure identified the MLP 6-10-14 architecture as the most suitable network structure for representing the dataset. The progressive increase in predictive accuracy with increasing numbers of neurons in the hidden layer followed by a plateau is consistent with the general behavior of ANN models in biological systems. When the number of neurons is too small, the network lacks sufficient flexibility to represent nonlinear relationships, whereas overly complex architectures tend to memorize the training data rather than capture the underlying process mechanisms. The plateau observed after approximately nine neurons therefore suggests that the essential structure of the dataset had already been learned by the network, and further complexity did not improve predictive capability. The high predictive performance observed for urease activity, ammonium production, ΔpH, and final pH indicates that the ANN model successfully reproduced the biochemical relationships associated with ureolytic metabolism. In ureolytic MICP systems, urea hydrolysis generates ammonium and carbonate species, increasing alkalinity and driving CaCO_3_ supersaturation. These reactions are tightly coupled and typically evolve in parallel during bacterial mineralization processes. Consequently, models trained on environmental parameters such as pH, nutrient concentration, and salinity often predict these variables with high accuracy because they follow well-defined biochemical pathways. Experimental investigations of MICP have similarly demonstrated that carbonate precipitation intensity closely tracks urease activity and the associated increase in pH within the local microenvironment [68,69].

The strong predictive capability of the ANN model for CaCO_3_ production further supports this interpretation. CaCO_3_ precipitation is the direct outcome of the ureolysis–alkalization cascade. Therefore, it remains closely linked to enzymatic activity and carbonate availability. Previous modeling studies of MICP systems have reported similar findings, where mineralization rates could be predicted effectively when urease activity and environmental conditions were incorporated into the model structure. These observations highlight that the precipitation process is primarily governed by chemical supersaturation dynamics generated by microbial metabolism rather than by purely stochastic biological variation [70]. Moderate predictive accuracy was obtained for parameters associated with bacterial growth dynamics, including bacterial concentration and survival rate. Such parameters often exhibit greater biological variability because they depend on physiological adaptation, metabolic regulation, and stress responses that occur at the cellular level. Environmental stressors such as salinity or nutrient limitation may influence bacterial populations through complex regulatory mechanisms that cannot be completely captured by simplified environmental descriptors. Experimental studies have shown that microbial growth and survival during biomineralization depend on substrate availability and other factors such as oxygen availability, ionic strength, and temperature, which collectively influence bacterial physiology and attachment behavior [71].

The lowest predictive accuracy in the present model was observed for lag time, μ_max_, aggregation, and average crystal size. These parameters represent emergent properties arising from interactions occurring across multiple spatial scales. For example, lag time reflects the physiological adjustment period required for cells to activate metabolic pathways under new environmental conditions. Similarly, crystal size and aggregation behavior are influenced by nucleation density, EPS, and cell clustering within microenvironments. Studies investigating the microstructure of MICP systems have demonstrated that bacterial density and local microenvironmental conditions strongly affect both the number and morphology of CaCO_3_ crystals formed during biomineralization [72]. Despite the variability observed for these parameters, the ANN model maintained consistent predictive performance across training, testing, and validation datasets. The similarity of *R*^2^ values across these subsets indicates that the model did not suffer from substantial overfitting and retained acceptable generalization ability. Statistical validation further confirmed the reliability of the predictions, as the mean bias error values remained close to zero and the distribution of residuals approached normality. These findings indicate that the network captured the dominant patterns present in the dataset while maintaining stability when applied to unseen data. The strong predictive performance observed for variables associated with ureolytic metabolism suggests that machine learning approaches can provide valuable insights into biomineralization systems. Recent research has increasingly emphasized the potential of data-driven modeling for predicting complex microbial processes, particularly when multiple interacting variables are involved. Numerical and computational models have been used to simulate MICP reactions in geotechnical and environmental systems, demonstrating that combining experimental data with advanced modeling tools can improve the prediction of CaCO_3_ precipitation dynamics and optimize process conditions [70,73]. The ability to predict CaCO_3_ precipitation and metabolic activity under varying environmental conditions is particularly relevant for the design of microbial self-healing materials [4]. In such systems, the efficiency of crack healing depends on both the rate and the localization of mineral formation. Predictive models capable of estimating biomineralization performance can therefore assist in identifying optimal conditions for bacterial activity, nutrient supply, and environmental stability. Machine learning tools such as ANN models are increasingly recognized as useful complements to experimental approaches in biotechnology and bio-based construction materials, enabling rapid exploration of multidimensional parameter spaces that would otherwise require extensive experimental campaigns. The ANN modeling results confirm that ureolysis-driven biochemical processes represent the dominant drivers of CaCO_3_ precipitation in the studied system. At the same time, the moderate predictability of growth kinetics and surface-related parameters reflects the inherent complexity of microbial behavior in biomineralization environments. By integrating experimental data with machine learning approaches, the present study demonstrates that predictive modeling can provide valuable insights into the optimization of microbial self-healing systems and other biomineralization-based technologies.

Despite the observed advantages of the UV-strain, several alternative interpretations and limitations should be considered. First, although enhanced urease activity and CaCO_3_ precipitation are commonly interpreted as indicators of improved self-healing potential, recent studies suggest that the spatial distribution, adhesion, and morphology of precipitated minerals may be more critical for crack sealing efficiency than the absolute quantity of CaCO_3_ produced. In this context, the relatively modest increase in CaCO_3_ yield observed between the strains may not necessarily translate into significantly improved performance in real cementitious systems. The reliance on ureolytic MICP introduces potential environmental and practical constraints, particularly due to ammonium accumulation as a metabolic by-product. Alternative non-ureolytic pathways, which avoid ammonium production, have been proposed as more sustainable options, although often with slower kinetics. Additionally, the experimental system employed in this study represents a controlled laboratory environment, which does not fully replicate the complexity of cementitious materials, including limited nutrient availability, pore structure constraints, and mechanical stresses. Therefore, the observed improvements in biomineralization performance should be interpreted as indicative rather than directly predictive of in situ self-healing efficiency. Finally, ANN modeling demonstrated strong predictive capability for key metabolic parameters, but the lower predictive accuracy for growth-related and surface properties highlights the inherent biological variability of these processes. This suggests that further integration of additional descriptors, such as extracellular polymeric substances or microenvironmental heterogeneity, may be necessary for fully mechanistic modeling of biomineralization systems.

The economic feasibility of bacteria-based self-healing systems remains an important consideration for large-scale application. Although the present study is limited to laboratory-scale evaluation, the main cost drivers include bacterial cultivation and spore production, nutrient supply, carrier materials, and delivery or encapsulation processes, with nutrients and delivery systems representing the dominant cost components. Compared to conventional repair techniques, such as epoxy injection or surface sealing, bio-based approaches may involve higher initial costs; however, they offer potential long-term economic advantages through autonomous crack repair, reduced maintenance, and extended service life, particularly in high-demand environments. The UV-induced phenotypic adaptation applied in this study represents a cost-effective strain improvement strategy, as it requires minimal equipment and avoids regulatory constraints associated with genetically modified organisms. Nevertheless, uncertainties remain regarding scalability potentially raising operational costs. In addition, the efficiency of bacterial activation and mineralization under real conditions will ultimately determine the cost–benefit balance. Therefore, future work should incorporate techno-economic analysis and life-cycle assessment to better evaluate the practical viability of such self-healing systems.

## 5. Conclusions

The main findings of this study can be summarized as follows:

• The UV-strain demonstrated enhanced biomineralization potential compared to the N-strain, with higher urease activity (approx. 33% increase), ammonium production, and CaCO_3_ precipitation (up to 2.37 mg/100 mL).
• Despite improved metabolic performance, the UV-strain retained comparable physiological robustness, with only minor reductions in survival under alkaline and saline conditions relevant to cementitious environments.
• Multivariate analysis confirmed that self-healing potential is governed by two key domains: (i) ureolysis-driven biomineralization efficiency, and (ii) physiological and surface-related properties influencing nucleation and mineral deposition.
• ANN modeling (MLP 6-10-14) demonstrated high predictive capability (*R*^2^ > 0.90) for key biomineralization parameters, confirming the suitability of data-driven approaches for modeling complex microbial systems.
• The results indicate that UV-induced phenotypic adaptation can be used as a simple and cost-effective strategy to enhance microbial performance for self-healing applications without significantly compromising its/their stability.
• The relatively small differences in CaCO_3_ yield and the controlled laboratory conditions highlight the need for caution when extrapolating results to real cementitious systems.
• The study introduces an integrated experimental modeling framework combining controlled UV-induced phenotypic adaptation with multivariate analysis and ANN modeling, enabling systematic evaluation and prediction of biomineralization performance in bacterial self-healing systems.

Future research should focus on the following: (i) evaluating the long-term stability and reproducibility of UV-induced phenotypic traits; (ii) validating the self-healing efficiency in real concrete and cement-based materials and under field-relevant conditions; (iii) optimizing nutrient systems to reduce ammonium-related environmental impacts; (iv) integrating techno-economic and life-cycle assessments to determine large-scale feasibility; as well as (v) improving predictive models by incorporating additional biological and microstructural parameters.

## Figures and Tables

**Table 1:** Response surface regression coefficients for ammonium-related response for N- and UV- *B. licheniformis* strains under varying pH, urea, NaCl, and incubation time conditions.

	N-Strain					UV-Strain				
Factor	Regr.Coeff.	Std.Err.	*p*	−95, %	+95, %	Regr.Coeff.	Std.Err.	*p*	−95, %	+95, %
Mean/Interc.	−115.24	32.81	0.001	−182.26	−48.23	−107.89	33.99	0.003	−177.30	−38.48
Time (L)	−0.29	0.13	0.040	−0.56	−0.01	−0.43	0.14	0.004	−0.71	−0.14
Time (Q)	0.00	0.00	0.733	0.00	0.00	0.00	0.00	0.308	0.00	0.00
pH initial (L)	28.35	6.73	0.000	14.61	42.09	27.61	6.97	0.000	13.38	41.84
pH initial (Q)	−1.60	0.35	0.000	−2.32	−0.89	−1.62	0.36	0.000	−2.37	−0.88
Urea (L)	−0.86	0.17	0.000	−1.22	−0.51	−0.79	0.18	0.000	−1.16	−0.43
Urea (Q)	0.00	0.00	0.071	0.00	0.00	0.00	0.00	0.717	0.00	0.00
NaCl (L)	−0.65	1.14	0.577	−2.98	1.69	−0.14	1.18	0.906	−2.56	2.28
NaCl (Q)	−0.05	0.04	0.200	−0.13	0.03	0.05	0.04	0.229	−0.03	0.13
Time × pH initial	0.02	0.01	0.052	0.00	0.05	0.05	0.01	0.001	0.02	0.07
Time × Urea	0.01	0.00	0.000	0.00	0.01	0.01	0.00	0.000	0.00	0.01
Time × NaCl	−0.01	0.00	0.215	−0.01	0.00	0.00	0.00	0.392	0.00	0.01
pH initial × Urea	0.08	0.02	0.000	0.04	0.11	0.08	0.02	0.000	0.05	0.12
pH initial × NaCl	0.12	0.11	0.301	−0.11	0.35	−0.04	0.12	0.726	−0.28	0.20
Urea × NaCl	0.01	0.01	0.247	0.00	0.02	0.00	0.01	0.999	−0.01	0.01
*R* ^2^	0.965					0.967				
adj.*R*^2^	0.949					0.952				

**Table 2:** Performance of the ANN Model Based on *R*^2^ values.

MLP 6-10-14	Bacterial Concentration	Survival Rate	Lag Time	µ_max_	Urease Activity	NH_4_	ΔpH	pH Final	CaCO_3_	Precipitation Rate	Average Crystal Size	Cell Electronegativity	Cell Hydrophobicity	Aggregation
Train	0.790	0.752	0.567	0.640	0.950	0.940	0.935	0.976	0.939	0.800	0.755	0.860	0.792	0.610
Test	0.708	0.787	0.583	0.580	0.906	0.924	0.945	0.983	0.919	0.584	0.629	0.894	0.765	0.603
Valid	0.803	0.554	0.658	0.588	0.927	0.857	0.883	0.971	0.875	0.693	0.474	0.705	0.686	0.648

**Table 3:** Verification results of the optimal ANN model.

Model	*χ* ^2^	RMSE	MBE	MPE	SSE	AARD	*r* ^2^	Skew	Kurt	Mean	StDev	Var
Bacterial concentration	0.046	0.215	−0.009	2.210	12.479	2.210	0.774	0.099	−0.183	−0.009	0.215	0.046
Survival rate	3.798	1.945	−0.228	1.699	1021.619	1.699	0.718	0.100	0.074	−0.228	1.935	3.746
Lag time	0.137	0.370	0.015	8.807	36.872	8.807	0.577	−0.053	−0.195	0.015	0.370	0.137
µ_max_	0.001	0.025	−0.001	3.831	0.164	3.831	0.620	−0.351	0.037	−0.001	0.025	0.001
Urease activity	0.497	0.704	0.063	8.459	133.796	8.459	0.943	0.174	0.188	0.063	0.702	0.493
NH_4_	2.092	1.444	0.085	10.121	562.882	10.121	0.928	0.096	0.098	0.085	1.444	2.085
ΔpH	0.020	0.141	0.010	11.887	5.404	11.887	0.928	0.202	0.099	0.010	0.141	0.020
pH final	0.023	0.150	0.014	1.110	6.059	1.110	0.976	0.130	0.232	0.014	0.149	0.022
CaCO_3_	75.643	8.681	0.067	15.010	20,347.890	15.010	0.929	0.048	−0.211	0.067	8.697	75.638
Precipitation rate	0.060	0.244	−0.002	15.650	16.033	15.650	0.756	−0.111	1.911	−0.002	0.244	0.060
Average crystal size	0.718	0.845	0.036	16.204	193.010	16.204	0.698	−0.053	0.156	0.036	0.846	0.716
Cell electronegativity	2.628	1.618	−0.035	−8.001	706.964	8.001	0.843	−0.052	0.022	−0.035	1.621	2.627
Cell hydrophobicity	8.434	2.899	0.202	9.336	2268.733	9.336	0.772	0.011	0.229	0.202	2.897	8.393
Aggregation	7.884	2.803	0.121	12.529	2120.884	12.529	0.608	−0.116	0.606	0.121	2.805	7.870

Note: chi-square statistic (*X*^2^), root mean square error (RMSE), mean bias error (MBE), mean per-centage error (MPE), sum of squared errors (SSE), average absolute relative deviation (AARD), coefficient of determination (*r*^2^), skewness (Skew), kurtosis (Kurt), mean (Mean), standard devar).iation (StDev), and variance (Var).

## Data Availability

The original contributions presented in the study are included in the article; further inquiries can be directed to the corresponding authors.

## Supplementary Materials

The following supporting information can be downloaded at: https://www.mdpi.com/article/10.3390/bioengineering13050495/s1, Table S1: Box–Behnken design; Table S2: Database set.
