# Supplementary material for: Ready-to-Use or Ready-to-Adapt: Can the Self-Healing Potential of Bacillus licheniformis Be Modified?
Source: Bioengineering (Basel). 2026 Apr 24;13(5):495. doi: 10.3390/bioengineering13050495 (PMC13203228; doi:10.3390/bioengineering13050495)
Supplement: Supplementary file 1 [file bioengineering-13-00495-s001.zip › Table S1.pdf]

Supplement Table S1: Box-Behnken design of experiment.

Run	pH	Urea	NaCl	pH	Urea (g/L)	NaCl (%)
1	-1	-1	0	8.5	10	4
2	-1	1	0	8.5	50	4
3	1	-1	0	10.5	10	4
4	1	1	0	10.5	50	4
5	-1	0	-1	8.5	30	1
6	-1	0	1	8.5	30	7
7	1	0	-1	10.5	30	1
8	1	0	1	10.5	30	7
9	0	-1	-1	9.5	10	1
10	0	-1	1	9.5	10	7
11	0	1	-1	9.5	50	1
12	0	1	1	9.5	50	7
13	0	0	0	9.5	30	4
14	0	0	0	9.5	30	4
15	0	0	0	9.5	30	4
